# Human *Escherichia coli* O157:H7 Genetic Marker in Isolates of Bovine Origin

**DOI:** 10.3201/eid1008.030784

**Published:** 2004-08

**Authors:** Jeffrey T. LeJeune, Stephen T. Abedon, Kaori Takemura, Nicholas P. Christie, Srinand Sreevatsan

**Affiliations:** * Ohio State University, Wooster, Ohio, USA

**Keywords:** E. coli O157, Shiga-toxin production, *stx*_2_-encoding phages, dispatch

## Abstract

The antiterminator *Q* gene of bacteriophage 933W (*Q*_933_) was identified upstream of the *stx*_2_ gene in 90% of human disease–origin *Escherichia coli* O157:H7 isolates and in 44.5% of bovine isolates. Shiga toxin production was higher in *Q*_933_-positive isolates than *Q*_933_-negative isolates. This genetic marker may provide a useful molecular tool for epidemiologic studies.

*Escherichia coli* O157 is recognized worldwide as an important cause of diarrheal disease, which in some patients is followed by hemolytic uremic syndrome and death (1). A primary virulence factor of this pathogen is the prophage-encoded Shiga toxin (2). Greater Shiga toxin production per bacterium is associated with increasing severity of human disease (3,4). Because of its location in the phage genome, the *stx*-gene variant dubbed *stx*_2_ is under similar regulatory control as other phage late-genes, as it is governed by the interaction of the transcription antiterminator Q with the late promoter P_R_´ (5).

Although cattle and other ruminants appear to be the natural reservoir for *E. coli* O157 and other Shiga toxin–producing *E. coli* (STEC), only a small fraction of STEC serotypes routinely present in cattle are frequently isolated from human patients. Mounting evidence suggests that considerable genetic, phenotypic, and pathogenic diversity exists among these pathogens (6–8). Furthermore, genetic subtypes or lineages of *E. coli* O157 do not appear to be equally distributed among isolates of bovine and human origin (7). The purpose of this study was to examine the distribution of specific sequences upstream of the *stx*_2_ gene among *E. coli* O157:H7 of human and bovine origin, along with corresponding magnitudes of Shiga toxin production.

## The Study

A total of 158 *stx*_2_-encoding *E. coli* O157:H7 isolates were assayed, 91 isolates of bovine origin and 67 originally isolated from ill persons (Tables A1 and A2). All isolates demonstrated unique banding patterns on pulsed-field gel electrophoresis (PFGE). For polymerase chain reaction (PCR) analysis, 5 µL of DNA obtained from boiled stationary-phase bacteria was added to a 50-µL PCR master mix containing a final concentration of 1.5 (*Q*_933_) or 2.5 (*Q*_21_) mmol MgCl_2_, 200 µmol/L each deoxynucleoside triphosphate, 1 U *Taq* polymerase, 0.6 pg/µL of primer 595 (5´-CCGAAGAAAAACCCAGTAACAG-3´) (9), and 0.6 pg/µL of either primer *Q*_933_ (5´-CGGAGGGGATTGTTGAAGGC-3´;Q_Stxf_) (9) or primer *Q*_21_ (5´-GAAATCCTCAATGCCTCGTTG-3´; this study). PCR consisted of an initial denaturation at 94°C for 5 min; 30 cycles of 94°C for 30 s, 52°C (*Q*_933_) or 55°C (*Q*_21_) for 1 min, and 72°C for 1 min; and a final 10-min extension step at 72°C. *E. coli* strain 933 or FAHRP88 was used as a positive control and master mix alone as a negative control. All PCR products were separated by gel electrophoresis (100 V) in 1% agarose gels, stained with ethidium bromide, and visualized by using UV illumination.

Shiga toxin production was determined by using a commercially available enzyme-linked immunosorbent assay (ELISA) kit (Premiere EHEC, Meridian Diagnostics, Cincinnati, OH). Briefly, log-phase cells from Luria-Bertani broth enrichments were diluted to 0.6 optical density (OD) at 600 nm, subsequently pelleted, resuspended in phosphate-buffered saline, and induced by exposure to UV light (240 nm) for 3 s (10). A 1:9 volume of a 10x concentrate of brain heart infusion broth was added to each culture and shaken at 37°C for 2.5 h. Replicate cultures that were not exposed to UV light (noninduced controls) were maintained at 4°C. Two hundred microliters of each induced and noninduced enrichment was subsequently used as the specimen in the EHEC ELISA, as described (11). OD results were recorded for each isolate both with and without UV induction. The relative change in Shiga toxin production after induction was calculated for each isolate; (OD_induced_)/OD_noninduced_). *E. coli* O157 (EDL933) and a toxin-negative control isolate were assayed as positive and negative controls each time the assay was repeated.

*E. coli* O157 isolates were classified on the basis of the presence or absence of bands of the predicted size on the *Q*_933_-595 and *Q*_21_-595 PCR reactions (Figure). A chi-square test was used to determine whether different PCR genotypes were equally distributed among isolates of bovine and human origin. Likewise, a chi-square test was used to assess the equality of distribution of PCR genotypes among bovine isolates from different countries. One-way analysis of variance for nonparametric data (Kruskal-Wallis test) was used to identify differences in ranked-transformed toxin production among noninduced and induced *E. coli* O157 isolates as well as to determine significant differences in the percent increase in toxin following induction.

**Figure:**
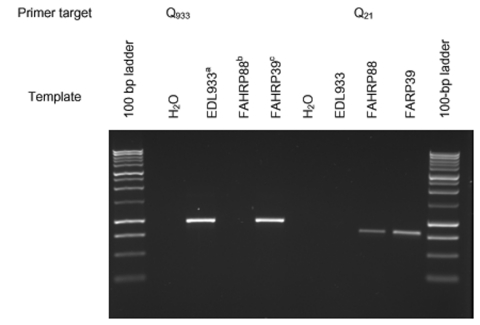
Ethidium bromide–stained gel of the amplification products obtained from Q933-595 and Q21-595 polymerase chain reactions. ^a^EDL933, human isolate (ATCC43895). Obtained from the STEC Center, Michigan State University. ^b^FAHRP88, isolated from Ohio dairy cow. ^c^FAHRP39, human isolate (E29962) (12).

Previously, Kim et al. described a nonrandom distribution of *E. coli* O157 subtypes among cattle and humans by using an octamer-based genome-scanning method (7). We tested several of the isolates that had been previously characterized. Nine had been previously identified as belonging to the lineage I genotype and seven isolates as belonging to the lineage II genotype. We found that all nine lineage I isolates consistently amplified the *Q*_933_ target, regardless of species of origin. All four bovine isolates classified as lineage II by Kim et al. amplified the *Q*_21_ target. One lineage II human isolate (NE015) amplified the *Q*_933_ target, and another lineage II isolate (NE037) produced no amplicons in either PCR reaction. One human isolate classified as lineage II (ATCC 43889) amplified both target sequences, presumably because of polylysogeny.

The distribution of the specific *Q*-gene alleles found upstream of the prophage *stx* region among bovine isolates may have a geographic component. The distribution of *E. coli* O157 phage genotypes collected from healthy cattle from diverse geographic areas is consistent with the variable incidences of human disease in different countries (Table 1). For example, six (75%) of eight Scottish bovine isolates examined amplified the *Q*_933_ target, the same target that is frequently present in human isolates of human disease origin. Scotland reports some of the highest incidence rates of human *E. coli* O157–related diseases and hemolytic uremic syndrome (13). In contrast, none of the seven Australian *E. coli* O157 bovine isolates amplified the 1750-bp fragment. Contrary to the situation in Scotland and the United States, *E. coli* O157 infection of humans is rarely reported in Australia (14).

**Table 1:** Distribution of polymerase chain reaction results from bovine *Escherichia coli* O157 isolates based on geographic origin^a^

Country of origin	No. tested	*Q* allele		
		933	21	Both
		N (%)	N (%)	N (%)
USA	46	20 (44)	25 (54)	1 (2)
Scotland	8	– (0)	2 (25)	6 (75)
Australia	7	– (0)	7 (100)	– (0)
Japan	17	3 (18)	14 (82)	– (0)
Total	78	23 (29)	48 (62)	7 (9)

^a^–, not detected. Percentages are read across rows, not down columns. Significant difference in proportion of *Q* alleles isolated from different countries (p < 0.05, chi-square test for homogeneity).

## Conclusions

The *Q*_933_ gene target was more commonly identified in human disease–associated strains of *E. coli* O157 than from strains of bovine origin. Amplification of the *Q*_933_ target, either alone or in combination with amplification of the *Q*_21_ target from the same isolate, was identified in 60 (9%) of 66 (55/66 alone and 5/66 in combination with *Q*_21_; 1 isolate amplified neither target) compared to 40 (44%) of 91 (32/91 alone, and 8/91 in combination with *Q*_21_) of bovine isolates (p < 0.001). Furthermore, these genetic subtypes were nonrandomly distributed among the *E. coli* O157 isolates of bovine origin obtained from different countries (p < 0.05) (Table 1).

These limited data suggest that the distribution of *E. coli* O157 strains in cattle may differ between countries or regions, thereby providing an explanation for geographic differences in the incidence of human *E. coli* O157 infection. More isolates from cattle need to be analyzed with these methods to better characterize the *E. coli* O157 in the bovine reservoir of each country.

A positive reaction with the *Q*_933_ target was significantly associated with higher OD results on the Shiga toxin ELISA (both noninduced and induced) and higher-fold increases in toxin production following induction than isolates amplifying the *Q*_21_ target alone (p < 0.0001) (Table 2). Despite these differences, we did not identify any clinical associations between the magnitude of Shiga toxin production and severity of human disease could be identified in this study. Other, non–Shiga toxin–related virulence factors and host susceptibility are also believed to play essential roles in the outcome of clinical STEC infections. The *Q*_933_-negative isolates obtained from human disease might have lost this *Q*_933_-containing prophage by the time of isolation, or these isolates might have been recovered from patients also infected with STEC containing *Q*_933_-type prophage (15). Whether specific *Q*-gene alleles directly correlate with the magnitude of Shiga-toxin production or whether other (unstudied) factors within the phage lytic cascade genetically linked to specific *Q* alleles instead are responsible for the magnitude of toxin production is not known.

**Table 2:** Shiga toxin production by *Escherichia coli* O157:H7 by *Q* allele

Assay	*Q* allele	Response		
		Median	Minimum	Maximum
OD_600nm_ noninduced	*Q*_933_	0.442	0.153	2.814
	*Q*_21_	0.170	0.120	0.413
OD_600nm_ induced	*Q*_933_	1.228	0.172	2.896
	*Q*_21_	0.165	0.084	1.210
Fold increase in OD_600nm_ after induction^a^	*Q*_933_	2.2	0.3	7.7
	*Q*_21_	0.9	0.4	5.1

^a^(OD_induced_)/(OD_noninduced_). The maximum and minimum optical density readings at 600 nm listed in each row are not necessarily from the same isolate; therefore, the maximum- and minimum-fold increase cannot be calculated directly from the table.

The antiterminator Q, the protein product of the *Q* gene, and *P*_R´_, the late promoter, are reputed to be involved in regulating phage late-genes and, because of the location of *P*_R´_ in prophage genome, of Shiga toxin production as well (5). In *E. coli* O157 phage 933W (GenBank no. 9632466) and *E. coli* O157 *stx_2vhd_* (GenBank no. 15718404), the 359-bp sequence immediately upstream of the *stx_2_* gene is nearly identical (>95% nucleotide identity). However, further upstream of this area of identity, DNA sequences differ significantly. In *E. coli* O157 933W, this gene is identified as the antiterminator *Q* gene. In contrast, in *E. coli* O157 *stx_2vhd_* this area is occupied by a gene with >95% sequence identity with the antiterminator *Q* gene of bacteriophage 21 (gi 4539472). The *Q* gene of bacteriophage 21 does not share DNA sequence homology with the *Q* gene of bacteriophage 933W, and only 36% predicted amino acid homology. Since the *Q* gene is reputed to play an important role in regulating toxin production, our results provide a plausible explanation (differential regulation of Shiga toxin production) of why certain *E. coli* O157 genotypes are more commonly isolated from human patients (7).

## Appendix

**Table A1:** Source of human isolates used in this study^a^

FAHRP ID	Source ID	Country	Year	Clinical signs and symptoms	References or source
6	FRIK 528	USA	1998	Diarrhea	16
7	FRIK 579	USA	1998	Diarrhea	16
8	93-001	USA	1999	Hemorrhagic colitis	17
9	ATCC 35150	USA	1999	Hemorrhagic colitis	17
16	91671	USA	1999	Hemorrhagic colitis	17
17	ATCC 43889	USA	1999	Hemorrhagic colitis	17
18	NE 037	USA	1999	Hemorrhagic colitis	17
19	NE 15	USA	1999	Hemorrhagic colitis	17
39	E29962	UK	1991	NR	18
54	CL56	Canada	1991	NR	18
60	E32511	USA	2002	HUS	19
58	EDL933	USA	1982	Hemorrhagic colitis	20
126	02 5225	USA	2002	NR	Washington^b^
127	02 4857	USA	2002	NR	Washington
128	02 6776	USA	2002	NR	Washington
129	02 6579	USA	2002	NR	Washington
130	02 6546	USA	2002	NR	Washington
131	02 6722	USA	2002	NR	Washington
132	02 6598	USA	2002	NR	Washington
133	02 6696	USA	2002	NR	Washington
134	02 6791	USA	2002	NR	Washington
135	02 6829	USA	2002	NR	Washington
136	02 6755	USA	2002	NR	Washington
137	02 6644	USA	2002	NR	Washington
138	06 781	USA	2002	Diarrhea	Idaho^c^
139	06 852	USA	2002	NR	Idaho
140	06 854	USA	2002	Watery diarrhea, vomiting	Idaho
141	06 856	USA	2002	Diarrhea	Idaho
142	06 855	USA	2002	NR	
143	06 886	USA	2002	Diarrhea, abdominal pain	Idaho
144	06 889	USA	2002	Abdominal pain	Idaho
145	06 988	USA	2002	Gastrointestinal bleeding	Idaho
146	07 004	USA	2002	Bloody stool	Idaho
147	07 007	USA	2002	Bloody stool	Idaho
148	07 023	USA	2002	Bloody stool	Idaho
149	07 085	USA	2002	NR	Idaho
150	07 147	USA	2002	NR	Idaho
151	07 154	USA	2002	NR	Idaho
152	O2191230	USA	2002	Diarrhea	Ohio^d^
153	O2191229	USA	2002	Diarrhea	Ohio
154	O2191231	USA	2002	Diarrhea	Ohio
155	O2191294	USA	2002	Diarrhea	Ohio
156	O2190819	USA	2002	Diarrhea	Ohio
157	O2190864	USA	2002	Diarrhea	Ohio
158	O2191309	USA	2002	Diarrhea	Ohio
159	O2191311	USA	2002	Diarrhea	Ohio
160	O2191313	USA	2002	Diarrhea	Ohio
161	O2191361	USA	2002	Diarrhea	Ohio
162	O2191602	USA	2002	Diarrhea	Ohio
163	O2191624	USA	2002	Diarrhea	Ohio
164	O2191541	USA	2002	Diarrhea	Ohio
165	O2191546	USA	2002	Diarrhea	Ohio
166	O2191423	USA	2002	Diarrhea	Ohio
167	O2191509	USA	2002	Diarrhea	Ohio
168	O2191363	USA	2002	Diarrhea	Ohio
169	O2191364	USA	2002	Diarrhea	Ohio
170	O2191365	USA	2002	Diarrhea	Ohio
171	O2191366	USA	2002	Diarrhea	Ohio
172	O2190889	USA	2002	Diarrhea	Ohio
173	O2190893	USA	2002	Diarrhea	Ohio
174	O2191176	USA	2002	Diarrhea	Ohio
175	O2191177	USA	2002	Diarrhea	Ohio
176	O2191623	USA	2002	Diarrhea	Ohio
177	O2191625	USA	2002	Diarrhea	Ohio
178	O2191645	USA	2002	Diarrhea	Ohio
179	O2191675	USA	2002	Diarrhea	Ohio
180	O2191765	USA	2002	Diarrhea	Ohio
181	O2191831	USA	2002	Diarrhea	Ohio

^a^FAHRP, Food Animal Health Research Program, Ohio State University; NR, not reported; HUS, hemolytic uremic syndrome. ^b^Washington State Department of Health isolates. ^c^Idaho Department of Health and Welfare isolates. ^d^Ohio Department of Health isolates.

**Table A2:** Source of bovine isolates used in this study

FAHRP^a^ ID	Source ID	Country	Year	References or source
1	FRIK 1986	USA	1991	21
2	FRIK 1997	USA	1991	21
3	FRIK 1994	USA	1991	21
4	FRIK 2002	USA	1991	21
5	FRIK 1987	USA	1991	21
10	FRIK 920	USA	1998	22
11	FRIK 1054	USA	1998	22
12	FRIK 1540	USA	1998	22
13	FRIK 1988	USA	1998	21
22	LCDC 87-2930	Canada	1991	23
27	OARDC1	USA	2002	FAHRP
29	OARDC2	USA	2002	FAHRP
31	OARDC3	USA	2002	FAHRP
35	P673	UK	1987	24
37	P277	UK	1987	24
47	c1526-77	Argentina	1991	23
50	CDC B9253-DMS1	USA	1991	23
51	A39	Canada	1991	23
52	A43	Canada	1991	23
56	LCDC 87-2924	Canada	1991	23
57	LCDC 87-1799	Canada	1991	23
62	CDC B6830-MS1/0	USA	1991	23
63	CDCB7205-MS1/0	USA	1991	23
64	CDC B8038-MS1/0	USA	1991	23
65	8832	USA	2002	25
66	EC66	USA	2002	FAHRP
67	EC 67	USA	2002	FAHRP
82	8833	USA	2002	25
83	EC 83	USA	2002	FAHRP
84	EC 84	USA	2002	FAHRP
85	8834	USA	2002	25
87	EC87	USA	2002	FAHRP
88	EC88	USA	2002	FAHRP
93	EC 93	USA	2002	FAHRP
94	EC94	USA	2002	FAHRP
95	EC95	USA	2002	FAHRP
96	EC96	USA	2002	FAHRP
97	EC97	USA	2002	FAHRP
98	EC98	USA	2002	FAHRP
99	EC99	USA	2002	FAHRP
100	EC100	USA	2002	FAHRP
102	EC102	USA	2002	FAHRP
103	EC103	USA	2002	FAHRP
104	EC104	USA	2002	FAHRP
113	8837	USA	2002	25
115	EC115	USA	2002	FAHRP
116	EC116	USA	2002	FAHRP
117	EC117	USA	2002	FAHRP
120	EC120	USA	2002	FAHRP
122	EC122	USA	2002	FAHRP
182	757	USA	1994	25
183	817	USA	1994	25
185	1104	USA	1994	25
186	1119	USA	1994	25
187	1124	USA	1994	25
188	1136	USA	1994	25
189	1273	USA	1994	25
190	3735	USA	1996	25
191	4048	USA	1996	25
192	7407	Japan	1996	25
193	7409	Japan	1996	25
194	7416	Japan	1996	25
195	7420	Japan	1996	25
196	7421	Japan	1996	25
197	7423	Japan	1996	25
198	7433	Japan	1996	25
199	7436	Japan	1996	25
200	7439	Japan	1996	25
201	7460	Japan	1996	25
202	7469	Japan	1996	25
203	7478	Japan	1996	25
204	7484	Japan	1996	25
205	7488	Japan	1996	25
206	7495	Japan	1996	25
207	7500	Japan	1996	25
208	7505	Japan	1996	25
209	7622	Scotland	1996	25
210	7630	Scotland	1999	25
211	7632	Scotland	1999	25
213	7637	Scotland	1999	25
214	7638	Scotland	1999	25
217	7648	Scotland	1999	25
218	7649	Scotland	1999	25
219	7653	Scotland	1999	25
220	8176	Australia	1999	25
221	8177	Australia	1996	25
222	8179	Australia	1997	25
223	8182	Australia	1997	25
224	8183	Australia	1997	25
225	8184	Australia	1998	25
226	8185	Australia	1999	25

^a^FAHRP, Food Animal Health Research Program, Ohio State University.
